# Effect of Liver Metastases on Survival in Microsatellite-Stable Metastatic Colorectal Cancer Treated with Immune Checkpoint Inhibitors

**DOI:** 10.1158/2767-9764.CRC-25-0690

**Published:** 2026-02-18

**Authors:** Nussara Pakvisal, Leontios Pappas, Bennett A. Caughey, Nora K. Horick, Noelia Tarazona, Kruti B. Vora, Ben Ouyang, Aditya Pandey, Bryan L. Peacker, Joie Sun, Leyre Zubiri, Kerry L. Reynolds, David P. Ryan, Motaz Qadan, Ryan B. Corcoran, Bruce Giantonio, Jill N. Allen, Elizabeth P. Walsh, Jefferey W. Clark, David T. Ting, Aparna R. Parikh

**Affiliations:** 1 Division of Hematology/Oncology, Mass General Brigham, Harvard Medical School, Boston, Massachusetts.; 2 Division of Medical Oncology, Department of Medicine, Faculty of Medicine, The King Chulalongkorn Memorial Hospital, Chulalongkorn University, Bangkok, Thailand.; 3 Mass General Brigham Cancer Center, Massachusetts General Hospital, Boston, Massachusetts.; 4 Department of Medical Oncology, INCLIVA Biomedical Research Institute, University of Valencia, Valencia, Spain.; 5 CIBERONC, Carlos III Health Institute, Madrid, Spain.; 6 Department of Medical Oncology, Dana-Farber Cancer Institute, Boston, Massachusetts.; 7 Division of Surgical Oncology, Department of Surgery, Massachusetts General Hospital, Boston, Massachusetts.

## Abstract

Immune checkpoint inhibitors (ICI) have limited efficacy in microsatellite-stable (MSS) metastatic colorectal cancer (mCRC), potentially because of immunosuppressive mechanisms associated with liver metastases. The impact of liver metastases on survival outcomes was evaluated in a retrospective cohort of patients with MSS mCRC treated with ICI-based therapies at Mass General Brigham between January 2015 and December 2022. Patients were stratified by liver metastasis status at ICI initiation. The primary endpoint was progression-free survival (PFS); the secondary endpoint was overall survival (OS). A total of 132 patients were included, of whom 93 (70.5%) had liver metastases at ICI initiation. Most patients in both groups had received ≥2 prior lines of therapy. No significant differences were observed between groups for *RAS/BRAF* mutation status or tumor mutational burden. Patients without liver metastases demonstrated higher clinical benefit rates (46.2% vs. 16.1%; *P* = 0.001), longer median PFS (2.5 vs. 2.1 months; HR, 1.68; *P* = 0.009), and higher 12-month PFS rates (12.8% vs. 1.1%; *P* = 0.034). The median OS was also prolonged in patients without liver metastases (11.5 vs. 6.2 months; HR, 2.03; *P* < 0.001). No history of liver metastases was an independent favorable risk factor for PFS and OS in univariable and multivariable analyses. These findings indicate that liver metastases are associated with inferior survival outcomes in patients with MSS mCRC treated with ICI-based therapies, supporting the immunosuppressive role of liver metastases and underscoring the importance of stratifying patients by liver metastasis status to guide patient selection and optimize therapeutic strategies.

**Significance:**

The limited efficacy of ICIs in MSS mCRC remains a major challenge. The association between liver metastases and inferior outcomes supports the liver’s immunosuppressive role and suggests that liver metastasis status may guide patient selection and treatment optimization.

## Introduction

Colorectal cancer is one of the most common malignancies worldwide and remains a leading cause of cancer-related mortality (1). Approximately 20% of patients present with metastatic disease [metastatic colorectal cancer (mCRC)] at diagnosis (2), and a significant proportion develop distant metastases during the disease course, with a 5-year survival rate of only 15% (3).

Immune checkpoint inhibitors (ICI) have transformed the treatment landscape for microsatellite instability–high (MSI-H)/deficient mismatch repair (dMMR) mCRC, producing durable responses (4, 5). In KEYNOTE-177, first-line pembrolizumab achieved a median progression-free survival (PFS) of 16.5 months (6), whereas CheckMate 8HW showed that nivolumab plus ipilimumab further prolonged PFS compared with nivolumab alone (not reached vs. 39.3 months) after a median follow-up of 47 months (7).

In contrast, microsatellite-stable (MSS)/proficient mismatch repair (pMMR) mCRC, representing approximately 95% of cases, has shown limited benefit from ICIs (8), even with combination strategies (9). Current first-line treatment with chemotherapy plus anti-EGFR or anti-VEGF therapies (3) provides disease control for 10 to 13 months (10–12), whereas later-line regimens such as regorafenib or trifluridine/tipiracil with bevacizumab offer only modest survival benefit (13–15), underscoring the unmet need for more effective strategies in this population.

MSS mCRC is characterized by an immune-excluded phenotype, with low tumor mutational burden (TMB) and scarce tumor-infiltrating lymphocytes (8). The presence of liver metastases, observed in up to 70% of patients (16), may further exacerbate immunosuppression (17). The liver’s intrinsic immune tolerance dampens cytotoxic T-cell and NK-cell activity (18), reinforced by Kupffer cells, regulatory T cells, and tumor-intrinsic mechanisms such as MHC-I downregulation and *B2M* mutations (17).

Although these mechanisms suggest that liver metastases could impair ICI efficacy, robust clinical evidence in MSS mCRC is lacking. To address this gap, we evaluated the association between liver metastases and survival outcomes in a long-term, real-world MSS mCRC cohort treated with ICI-based regimens, with the goal of assessing its potential as a clinical biomarker to guide patient selection and optimize treatment strategies.

## Materials and Methods

### Patient population

We retrospectively reviewed medical records of patients with mCRC treated at Mass General Brigham Cancer Center between January 2015 and December 2022. Eligible patients had MSS or pMMR tumors and received ICI-based therapy, defined as ICI monotherapy (anti–PD-1/–PD-L1), dual ICI therapy (anti–PD-1/–PD-L1 plus anti–CTLA-4), or ICI combined with other systemic treatments, regardless of treatment line.

ICI-based therapy is not considered a standard treatment for MSS/pMMR tumors, either during the study period or at present. Therefore, patients were eligible for inclusion if they received investigational ICI-based regimens as part of clinical trials or through individualized treatment decisions made by the treating physician. Cases with known POLE mutations were excluded because of their distinct molecular profile and ICI responsiveness, which resembles MSI-H disease (19).

In the primary analysis, patients were categorized by liver metastasis status at ICI initiation: with liver metastases or without. The latter group included patients with previously treated liver metastases and no radiographic evidence of disease at ICI initiation. For Cox regression analysis, liver status was further classified into three groups: (i) active liver metastases—radiologic or clinical evidence at ICI initiation, including residual or recurrent disease after resection; (ii) complete liver metastasectomy—prior metastases fully resected with no active disease; and (iii) no history of liver metastases—patients never diagnosed with liver metastases. Molecular alteration data were obtained from medical records, including results from all institutionally approved sequencing platforms and available sample sites.

### Endpoints

The primary endpoint of this study was PFS, comparing patients with and without liver metastases. PFS was defined as the time from ICI initiation to disease progression or death from any cause.

Secondary endpoints included overall survival (OS), defined as the time from ICI initiation to death from any cause, and clinical benefit rate (CBR), defined as the proportion of patients whose best response to ICI-based therapy was not progressive disease. In addition, we investigated clinical and molecular factors associated with PFS and OS in this study population.

### Statistical analysis

Comparisons of demographic and clinical characteristics between patients with and without liver metastases were conducted using *χ*^2^ tests for categorical variables and Student *t* tests for continuous variables.

Differences in PFS and OS between groups were assessed using two-sided log-rank tests, and survival curves were estimated using the Kaplan–Meier method. To evaluate the association between survival outcomes and liver metastasis status, as well as other factors, univariable Cox regression analysis was used to calculate unadjusted hazard ratios (HR). Multivariable models were then applied to adjust for potential confounders and assess the independent association of liver metastasis status with PFS and OS. A *P* value < 0.05 was considered statistically significant.

All statistical analyses were performed using SPSS version 28.0 (IBM Corp.).

This study was conducted in accordance with the Declaration of Helsinki and was deemed exempt by the Institutional Review Board of Mass General Brigham (approval number: 2017P000501); thus, the requirement for written informed consent was waived.

## Results

### Patient characteristics

**Figure 1.**
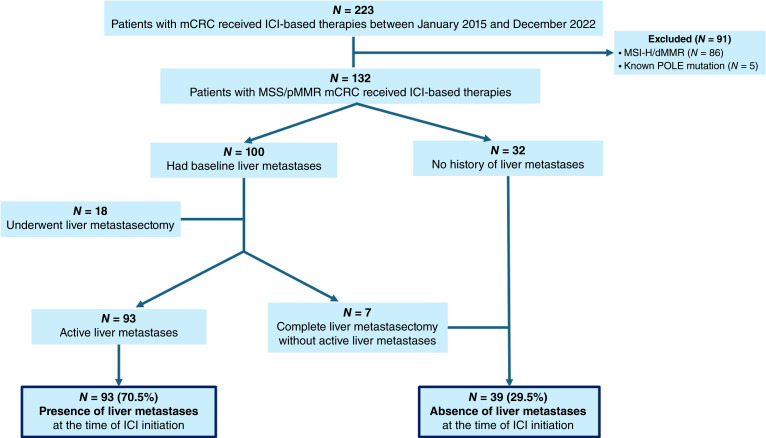
Consort flow diagram.

A total of 223 patients with mCRC who received ICI-based therapies were identified. Ninety-one patients were excluded (86 MSI-H/dMMR and five with *POLE* mutations). The final study cohort included 132 patients with MSS/pMMR mCRC. At baseline, 100 patients (75.8%) had liver metastases, including seven who had undergone complete liver metastasectomy with no radiographic evidence of disease. For the primary analysis, 93 patients (70.5%) were classified as having liver metastases at ICI initiation (Fig. 1).

Baseline demographics and clinical characteristics are summarized in Table 1. No statistically significant differences were observed in most baseline characteristics, except that patients with liver metastases had a higher rate of synchronous metastases (72% vs. 46%; *P* = 0.005). Patients without liver metastases showed nonsignificant trends toward more primary rectal tumors, peritoneal metastases, and bone metastases.

**Table 1.** Demographic and clinical characteristics of patients with MSS/pMMR mCRC by liver metastasis status.

Characteristic	All *N* = 132	Liver metastases *N* = 93	Without liver metastases *N* = 39	*P* value
Age at diagnosis of metastasis	​	​	​	​
Median (range), years	49	50	47	​
≥65 years, *n* (%)	16 (12.1)	13 (14)	3 (7.7)	0.313
Sex, *n* (%)	​	​	​	0.066
Male	77 (58.3)	59 (63.4)	18 (46.2)	​
Female	55 (41.7)	34 (36.6)	21 (53.8)	​
ECOG PS, *n* (%)	​	​	​	0.257
0–1	129 (97.7)	90 (96.8)	39 (100)	​
2	3 (2.3)	3 (3.2)	0 (0)	​
Sidedness, *n* (%)	​	​	​	0.758
Right sided	33 (25)	24 (25.8)	9 (23.1)	​
Left sided	98 (74.2)	68 (73.1)	30 (76.9)	​
Unknown	1 (0.8)	1 (1.1)	0 (0)	​
Tumor location, *n* (%)	​	​	​	0.061
Colon	99 (75)	74 (79.6)	25 (64.1)	​
Rectum	33 (25)	19 (20.4)	14 (35.9)	​
Metastatic type, *n* (%)	​	​	​	**0.005**
Synchronous	**85 (64.4)**	**67 (72)**	**18 (46.2)**	​
Metachronous	**47 (35.6)**	**26 (28)**	**21 (53.8)**	​
Other metastatic sites, *n* (%)	​	​	​	​
Distant lymph nodes	80 (60.6)	58 (62.4)	22 (56.4)	0.523
Lung	96 (72.7)	67 (72)	29 (74.4)	0.785
Peritoneum	29 (22)	18 (19.4)	11 (28.2)	0.263
Bone	25 (18.9)	16 (17.2)	9 (23.1)	0.432
Others	18 (13.6)	12 (12.9)	6 (15.4)	0.705
Molecular profile^a^, *n* (%)	​	​	​	​
*KRAS* mutation	78 (59.1)	53 (57)	25 (64.1)	0.448
*BRAF* mutation	9 (6.8)	7 (7.5)	2 (5.1)	0.618
*NRAS* mutation	8 (6.1)	7 (7.5)	1 (2.6)	0.276
*APC* mutation^b^	86 (65.2)	64 (68.8)	22 (56.4)	0.387
*TP53* mutation^c^	95 (72)	70 (75.3)	25 (64.1)	0.424
*PIK3CA* mutation^d^	26 (19.7)	17 (18.3)	9 (23.1)	0.193
HER2 overexpression^e^	5 (3.8)	2 (2.2)	3 (7.7)	0.284
None of the above	1 (0.8)	1 (1.1)	0 (0)	0.516
TMB, *n* (%)	​	​	​	0.222
Low (<10 mut/mb)	36 (27.3)	27 (29)	9 (23.1)	​
High (≥10 mut/mb)	40 (30.3)	24 (25.8)	16 (41)	​
Unknown	56 (42.4)	42 (45.2)	14 (35.9)	​
Previous lines of systemic Tx, *n* (%)	​	​	​	​
0	3 (2.3)	2 (2.2)	1 (2.6)	0.639
1	26 (19.7)	18 (19.4)	8 (20.5)	​
2	46 (34.8)	34 (36.6)	12 (30.8)	​
3	25 (18.9)	16 (17.2)	9 (23.1)	​
≥4	31 (23.5)	23 (24.7)	8 (20.5)	​
Unknown	1 (0.8)	0 (0)	1 (2.6)	​
Type of ICI, *n* (%)	​	​	​	0.879
ICI monotherapy	22 (16.7)	15 (16.1)	7 (17.9)	​
Dual ICI therapy	4 (3)	3 (3.2)	1 (2.6)	​
ICI combined with other Tx	106 (80.3)	75 (80.6)	31 (79.5)	​
Metastasectomy before ICI initiation, *n* (%)	​	​	​	0.306^f^
Yes	31 (23.5)	18 (19.4)	13 (33.3)	​
Liver	19 (14.4)	12 (12.9)	7 (17.9)	​
Lung	3 (2.3)	1 (1.1)	2 (5.1)	​
Peritoneum	6 (4.5)	4 (4.3)	2 (5.1)	​
Brain	1 (0.8)	0 (0)	1 (2.6)	​
Others	2 (1.5)	1 (1.1)	1 (2.6)	​
No	101 (76.5)	75 (80.6)	27 (69.2)	​
Subsequent Tx after ICI, *n* (%)	​	​	​	0.191
Yes	63 (47.7)	42 (45.2)	21 (53.8)	​
No	66 (50)	50 (53.8)	16 (41)	​
Unknown	3 (2.3)	1 (1.1)	2 (5.1)	​

Bold values indicate statistically significant results (*P* < 0.05).

Abbreviations: mut/mb, mutations per mega base; Tx, treatment.

a All patients underwent at least one molecular test at baseline or prior to ICI treatment.

b Data were missing for 17 cases (13%).

c Data were missing for 19 cases (14%).

d Data were missing for 23 cases (17%).

e Data were missing for 25 cases (19%).

f *P* value comparing history of metastasectomy versus no metastasectomy.

In terms of molecular characteristics, all patients underwent at least one molecular test either at baseline or prior to ICI initiation. Although no statistically significant differences were detected, distinct mutation patterns were observed between the groups. Patients with liver metastases more often had *APC* and *TP53* mutations, whereas those without had higher frequencies of *KRAS *mutations, HER2 overexpression, and high TMB. Unfortunately, nearly half of the total cohort lacked available TMB data. Additional details on molecular alterations are provided in Supplementary Table S1.

Most patients in both groups were heavily pretreated, with a median of two prior therapy lines. ICI-based therapies were predominantly given in combination with other systemic treatments, particularly novel targeted agents. A total of 76.5% were investigational regimens given in clinical trials (Supplementary Table S2). Among patients receiving post-ICI therapy, proportions were slightly higher in the non–liver metastasis group but not significantly different (Supplementary Table S3).

### Survival outcomes and CBRs

**Figure 2.**
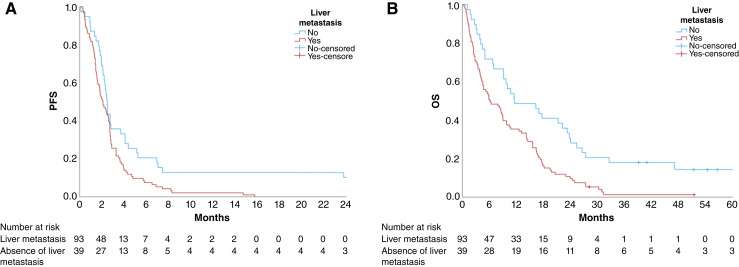
Kaplan–Meier curves for survival outcomes in patients with MSS/pMMR mCRC receiving ICI-based therapies, stratified by liver metastasis status at the time of ICI initiation. **A,** PFS: Patients without liver metastases had significantly higher 12-month PFS rates (12.8% vs. 1.1%; *P* = 0.034). The median PFS was 2.1 months (95% CI, 1.59–2.62) in those with liver metastases and 2.5 months (95% CI, 2.22–2.71) in those without liver metastases (HR, 1.68; 95% CI, 1.13–2.5; *P* = 0.009). **B,** OS: Median OS was 11.53 months (95% CI, 3.01–20.06) in those without liver metastases and 6.17 months (95% CI, 2.87–9.46) in those with liver metastases (HR, 2.03; 95% CI, 1.35–3.06; *P* < 0.001).

The primary endpoint PFS was significantly longer in patients without liver metastases at the time of ICI initiation, at 2.5 months [95% confidence interval (CI), 2.22–2.71] versus 2.1 months (95% CI, 1.59–2.62) in those with liver metastases (*P* = 0.009; Fig. 2A), with an HR of 1.68 (95% CI, 1.13–2.51). Patients without liver metastases also had a significantly higher CBR from ICI-based therapy compared with those with liver metastases (46.2% vs. 16.1%; *P* = 0.001). As of the data cutoff date, with a median follow-up of 51.5 months, five patients (12.8%) without liver metastases and two patients (2.2%) with liver metastases remained alive. The median OS was significantly prolonged in patients without liver metastases, reaching 11.53 months (95% CI, 3.01–20.06) compared with 6.17 months (95% CI, 2.87–9.46) in those with liver metastases (*P* < 0.001; Fig. 2B), with an HR of 2.03 (95% CI, 1.35–3.06).

Additionally, we compared PFS and OS between patients with and without liver metastases at the time of initial diagnosis. No statistically significant differences were observed, with an HR of 1.11 (95% CI, 0.74–1.67) for PFS and 1.38 (95% CI, 0.91–2.08) for OS (Supplementary Fig. S1A and S1B).

For sites of progressive disease during ICI-based therapy, the liver was the only site showing a significant difference between groups (86% in patients with liver metastases vs. 7.7% in those without; *P* < 0.001; Supplementary Table S4). Notably, among patients without liver metastases at the time of ICI initiation who had previously undergone complete liver metastasectomy, none experienced progression in the liver.

### Factors associated with survival outcomes

The univariable and multivariable Cox regression results for PFS and OS are presented in Tables 2 and 3, respectively. Using the active liver metastases group as the reference, patients with no history of liver metastases had significantly improved PFS and OS in both univariable and multivariable analyses. In contrast, complete liver metastasectomy was associated with better outcomes in univariable analysis but did not remain significant in the multivariable models.

**Table 2.** Univariable and multivariable Cox regression analysis for PFS.

Covariate	Univariable			Multivariable		
	HR	95% CI	*P* value	HR	95% CI	*P* value
Age ≥65 vs. <65 years	0.94	0.55–1.58	0.808	1.33	0.57–3.12	0.508
Male vs. female	1	0.71–1.42	0.976	1.10	0.57–2.15	0.776
ECOG PS ≥2 vs. 0–1	**5.31**	**1.63–17.33**	**0.006**	4.83	0.51–45.73	0.17
Left vs. right sided	0.83	0.55–1.24	0.351	0.98	0.42–2.23	0.95
Rectum vs. colon	0.89	0.59–1.32	0.552	0.72	0.33–1.58	0.416
Synchronous vs.	​	​	​	​	​	​
Metachronous metastasis	1.19	0.83–1.70	0.356	1.62	0.87–3	0.125
Liver metastasis status	​	​	​	​	​	​
Active liver metastases	1	​	​	1	​	​
Complete liver metastasectomy	0.77	0.51–1.16	0.207	1.51	0.70–3.23	0.290
No history of liver metastases	**0.18**	**0.06–0.52**	**0.002**	**0.04**	**0.006–0.23**	**<0.001**
Lymph node metastasis	​	​	​	​	​	​
Yes vs. no	1.06	0.75–1.51	0.741	1.28	0.66–2.50	0.467
Lung metastasis	​	​	​	​	​	​
Yes vs. no	1.14	0.77–1.68	0.527	1.33	0.62–2.85	0.465
Peritoneal metastasis	​	​	​	​	​	​
Yes vs. no	1.41	0.93–2.13	0.110	**2.40**	**1.04–5.54**	**0.041**
Bone metastasis	​	​	​	​	​	​
Yes vs. no	1.12	0.71–1.74	0.633	1.75	0.76–4	0.187
Combination Tx	​	​	​	​	​	​
vs. ICI alone	0.71	0.46–1.01	0.116	0.53	0.21–1.33	0.175
Previous systemic Tx	​	​	​	​	​	​
Yes vs. no	2.05	0.65–6.47	0.223	2.16	0.44–10.55	0.341
High vs. low TMB	1.35	0.85–2.15	0.208	1.03	0.46–2.31	0.941
*KRAS* mutation	​	​	​	​	​	​
Yes vs. no	1.16	0.82–1.65	0.408	0.92	0.47–1.79	0.801
*NRAS* mutation	​	​	​	​	​	​
Yes vs. no	0.83	0.40–1.69	0.600	0.74	0.21–2.62	0.646
*BRAF* mutation	​	​	​	​	​	​
Yes vs. no	0.87	0.44–1.72	0.692	2.07	0.66–6.54	0.214
*APC* mutation	​	​	​	​	​	​
Yes vs. no	1.06	0.69–1.62	0.809	1.99	0.89–4.48	0.096
*TP53* mutation	​	​	​	​	​	​
Yes vs. no	1.01	0.60–1.70	0.960	1.77	0.62–5.07	0.290
*PIK3CA* mutation	​	​	​	​	​	​
Yes vs. no	0.98	0.62–1.53	0.918	1.21	0.62–2.34	0.584
Her2 overexpression	​	​	​	​	​	​
Yes vs. no	0.40	0.14–1.14	0.087	2.19	0.47–10.26	0.341

Bold values indicate statistically significant results (*P* < 0.05).

Abbreviation: Tx, treatment.

**Table 3.** Univariable and multivariable Cox regression analysis for OS.

Covariate	Univariable			Multivariable		
	HR	95% CI	*P* value	HR	95% CI	*P* value
Age ≥65 vs. <65 years	1.38	0.81–2.33	0.234	1.25	0.48–3.23	0.648
Male vs. female	0.99	0.70–1.42	0.973	1.01	0.52–1.95	0.979
ECOG PS ≥2 vs. 0–1	**15.04**	**4.23–53.49**	**<0.001**	**10.04**	**1.18–85.73**	**0.035**
Left vs. right sided	0.81	0.54–1.21	0.303	0.72	0.30–1.76	0.470
Rectum vs. colon	0.84	0.56–1.27	0.412	0.72	0.30–1.72	0.455
Synchronous vs.	​	​	​	​	​	​
Metachronous metastasis	1.25	0.87–1.82	0.232	**2.70**	**1.33–5.48**	**0.006**
Liver metastasis status	​	​	​	​	​	​
Active liver metastases	**1**	​	​	**1**	​	​
Complete liver metastasectomy	**0.61**	**0.40–0.93**	**0.021**	**0.73**	**0.37–1.47**	**0.382**
No history of liver metastases	**0.16**	**0.05–0.50**	**0.002**	**0.006**	**0.00–0.07**	**<0.001**
Lymph node metastasis	​	​	​	​	​	​
Yes vs. no	1.16	0.78–1.60	0.553	0.55	0.28–1.07	0.076
Lung metastasis	​	​	​	​	​	​
Yes vs. no	0.95	0.64–1.41	0.790	**0.46**	**0.22–0.95**	**0.034**
Peritoneal metastasis	​	​	​	​	​	​
Yes vs. no	**1.61**	**1.05–2.46**	**0.028**	1.40	0.62–3.18	0.418
Bone metastasis	​	​	​	​	​	​
Yes vs. no	1.16	0.73–1.82	0.536	**3.46**	**1.40–8.56**	**0.007**
Combination Tx	​	​	​	​	​	​
vs. ICI alone	0.96	0.62–1.50	0.865	0.86	0.35–2.09	0.736
Previous systemic Tx	​	​	​	​	​	​
Yes vs. no	1.31	0.42–4.15	0.641	0.49	0.10–2.48	0.388
Subsequent Tx	​	​	​	​	​	​
Yes vs. no	**0.25**	**0.17–0.36**	**<0.001**	**0.07**	**0.03–0.17**	**<0.001**
High vs. low TMB	1.07	0.66–1.72	0.797	0.85	0.39–1.85	0.686
*KRAS* mutation	​	​	​	​	​	​
Yes vs. no	1.33	0.93–1.91	0.121	**2.67**	**1.31–5.46**	**0.007**
*NRAS* mutation	​	​	​	​	​	​
Yes vs. no	1.12	0.55–2.30	0.758	0.44	0.12–1.52	0.193
*BRAF* mutation	​	​	​	​	​	​
Yes vs. no	1.07	0.52–2.19	0.854	2.50	0.65–9.63	0.182
*APC* mutation	​	​	​	​	​	​
Yes vs. no	0.81	0.52–1.25	0.336	0.80	0.40–1.61	0.536
*TP53* mutation	​	​	​	​	​	​
Yes vs. no	1.03	0.60–1.75	0.916	0.62	0.23–1.69	0.350
*PIK3CA* mutation	​	​	​	​	​	​
Yes vs. no	0.84	0.53–1.34	0.469	1.50	0.78–2.89	0.223
Her2 overexpression	​	​	​	​	​	​
Yes vs. no	**0.31**	**0.10–0.97**	**0.044**	1.02	0.20–5.20	0.980

Bold values indicate statistically significant results (*P* < 0.05).

Abbreviation: Tx, treatment.

In the multivariable analysis, no history of liver metastases was independently associated with longer PFS, whereas peritoneal metastases were associated with worse PFS. For OS, favorable prognostic factors included no history of liver metastases, involvement of lung metastases, and receipt of subsequent systemic therapy. In contrast, worse OS was associated with Eastern Cooperative Oncology Group (ECOG) performance status (PS) of ≥2, synchronous metastases, bone metastases, and *KRAS* mutation.

## Discussion

ICIs have significantly improved survival outcomes in MSI-H/dMMR mCRC (4, 6, 7). In contrast, most patients with MSS/pMMR mCRC have limited treatment options that provide long-term disease control beyond 5-fluorouracil–based chemotherapy (20). In this population, ICIs alone have failed to demonstrate clinical benefit in MSS mCRC (20–22) although signals of efficacy are emerging in the population of patients without liver metastases (23). Various combination strategies have been explored to overcome immune resistance and improve outcomes, yet no regimen has demonstrated a survival benefit in a phase III clinical trial (9, 24). Identifying clinical biomarkers to guide patient selection is therefore essential. Our study directly addresses this gap by evaluating the impact of liver metastases on ICI efficacy in MSS mCRC.

We found that liver metastasis status at the time of ICI initiation, rather than at the time of initial diagnosis, was strongly associated with treatment benefit. Patients without liver metastases at ICI initiation experienced higher CBRs and longer survival. In contrast, when patients who had previously undergone complete liver metastasectomy were included in the liver metastasis group, there was no significant survival difference based on liver metastasis status at diagnosis. When we categorized patients into three groups, consisting of those with active liver metastases, those with prior complete liver metastasectomy, and those with no history of liver metastases, the best outcomes were observed in patients with no history of liver metastases. This association remained significant in multivariable models for both PFS and OS. These findings indicate that liver metastasis status at the start of ICI therapy is a clinically relevant predictor of benefit in MSS mCRC.

Our observations are consistent with subgroup data from two phase III trials—IMblaze370 (cobimetinib + atezolizumab) and LEAP-017 (lenvatinib + pembrolizumab), both of which failed to improve survival over regorafenib or trifluridine/tipiracil in unselected MSS mCRC (24, 25). Notably, ∼70% of enrolled patients in each study had liver metastases, which may have contributed to the lack of benefit (24, 25). In LEAP-017, patients without liver metastases showed a trend toward improved OS (HR, 0.65; 95% CI, 0.42–0.99), whereas those with liver metastases derived minimal benefit (HR, 0.91; 95% CI, 0.72–1.15; ref. 25). Our study expands on these findings by confirming the association across a broad range of ICI-based regimens, including many investigational combinations and real-world practice treatments.

The poor outcomes in patients with liver metastases suggest a biological basis for ICI resistance, likely driven by the immunosuppressive hepatic microenvironment, which hinders T-cell infiltration (26) as observed in other cancers (27, 28). In colorectal cancer, liver metastases exhibit three distinct histologic growth patterns—desmoplastic, replacement, and pushing—which further contribute to immune resistance (29). The desmoplastic pattern’s fibrotic rim forms a physical barrier; the replacement pattern co-opts vasculature to create immune-desert regions; and the pushing pattern, although less defined, may also facilitate immune evasion (29). In our study, we observed a tendency for higher prevalence of APC mutations in patients with liver metastases, suggesting that WNT/β-catenin pathway activation may promote T-cell exclusion as an additional resistance mechanism (30, 31). These mechanisms provide a rationale for the reduced ICI efficacy observed in patients with MSS mCRC with liver metastases, underscoring the need for alternative therapeutic strategies. Notably, Cox regression analysis indicated a potential survival benefit for patients who underwent complete liver metastasectomy and had no liver disease at ICI initiation compared with those with active liver metastases. Although this association did not reach statistical significance, likely because of the small sample size (*N* = 7), none of these patients experienced liver progression during ICI therapies. These findings propose that incorporating liver-directed interventions to achieve local disease control may enhance systemic immune responses and potentially improve outcomes in this population (32).

Additionally, the clinical relevance of liver metastasis status is further supported by phase I to II prospective clinical trials of immunotherapy-based strategies that have focused on patients with no active liver metastases, including combinations of vilastobart with atezolizumab (33), muzastotug with pembrolizumab (34), and botensilimab with balstilimab (35). These regimens have demonstrated objective response rates in the range of approximately 20%. Notably, the latter two combinations have received regulatory authorization to proceed to phase III evaluation, with randomized studies planned that are designed to enrich patients without active liver metastases (36, 37).

With regard to factors associated with survival in our study, liver metastasis status was the only variable significantly associated with PFS in both univariable and multivariable analyses. For OS, two additional factors—PS and receipt of subsequent treatment—also reached statistical significance. PS is a well-established prognostic factor in colorectal cancer (38) and multiple retrospective studies across tumor types have reported that patients with ECOG PS ≥2 experience lower response rates and worse OS with ICI therapy (39–41), consistent with our findings. In terms of subsequent treatment, a selection bias may exist as patients with better health status were more likely to receive additional systemic therapy. We accounted for this by including subsequent therapy as a covariate in the OS analysis. Ultimately, Cox regression analysis demonstrated that continuing systemic therapy after ICI progression significantly improved OS, underscoring the importance of treatment sequencing in MSS mCRC.

### Strengths and limitations

One of the major strengths of our study is the long-term follow-up cohort, with a median follow-up of 51.5 months in patients with MSS/pMMR mCRC treated with various ICI-based regimens. In addition, 80% of patients had comprehensive molecular profiling data available. This enabled a robust assessment of survival outcomes and predictive factors. To our knowledge, this is the first study to evaluate the association between OS benefit and liver metastasis status in patients with MSS/pMMR mCRC receiving ICI-based therapies, demonstrating that the presence of liver metastases is associated with worse PFS and OS. Additionally, we applied multivariable analysis adjusted for all potential confounders, further enhancing the reliability of our findings.

However, we acknowledge several limitations. First, this was a retrospective, single-system study, which may introduce selection biases and imbalances in baseline characteristics. To mitigate this, we conducted multivariable analyses as mentioned above. Second, the heterogeneity of ICI-based therapy, primarily due to investigational regimens in clinical trials, may have contributed to variable responses. Future prospective studies in larger cohorts could help address this limitation. Lastly, the presence of missing TMB data limited our ability to perform a more comprehensive assessment of the interaction between TMB, liver metastases, and ICI response. As previous studies have reported that TMB may not be a strong predictive biomarker for ICI efficacy and lacks validated cutoffs in mCRC (42–44), its absence is unlikely to have significantly affected the overall outcomes of this study.

### Conclusion

Although ICIs are not currently a standard treatment for MSS/pMMR mCRC, they continue to be used in real-world practice for refractory patients with limited therapeutic options. Drawing on one of the largest real-world MSS/pMMR mCRC cohorts to date, treated with both investigational combinations and physician-selected regimens, our study identifies the absence of liver metastases at ICI initiation as an independent predictor of favorable survival. This finding reinforces its potential role in patient selection and treatment stratification. A large prospective study is warranted to confirm these results and to optimize the integration of ICI-based regimens for this population. Conversely, the immune-tolerant liver microenvironment may contribute to ICI resistance in patients with liver metastases, highlighting the need to explore tailored immunotherapeutic approaches, including the integration of liver-directed treatments, to enhance efficacy in this subgroup.

## Supplementary Material

Supplementary Figure 1 Kaplan-Meier curves for survival outcomes in MSS/pMMR mCRC patients receiving ICI-based therapies, stratified by liver metastases status at baseline

Supplementary Table 1 Details of molecular characteristics and tumor mutational burden by timing of treatment

Supplementary Table 2 Regimens of immune checkpoint inhibitor-based therapies

Supplementary Table 3 Subsequent treatments after immune checkpoint inhibitor-based therapy

Supplementary Table 4 Sites of progressive disease during immune checkpoint inhibitor-based therapy

## Data Availability

The datasets analyzed during the study are available from the corresponding author on reasonable request.
