# Supplementary material for: Effect of Liver Metastases on Survival in Microsatellite-Stable Metastatic Colorectal Cancer Treated with Immune Checkpoint Inhibitors
Source: Cancer Res Commun. 2026 Feb 18;6(2):340–9. doi: 10.1158/2767-9764.CRC-25-0690 (PMC13038315; doi:10.1158/2767-9764.CRC-25-0690)
Supplement: Supplementary Figure 1 — Kaplan-Meier curves for survival outcomes in MSS/pMMR mCRC patients receiving ICI-based therapies, stratified by liver metastases status at baseline [file crc-25-0690_supplementary_figure_1_suppsf1.docx]

**Supplementary Figure1.** Kaplan-Meier curves for survival outcomes in MSS/pMMR mCRC patients receiving ICI-based therapies, stratified by liver metastases status at baseline: (A) Progression-Free Survival; (B) Overall Survival

**
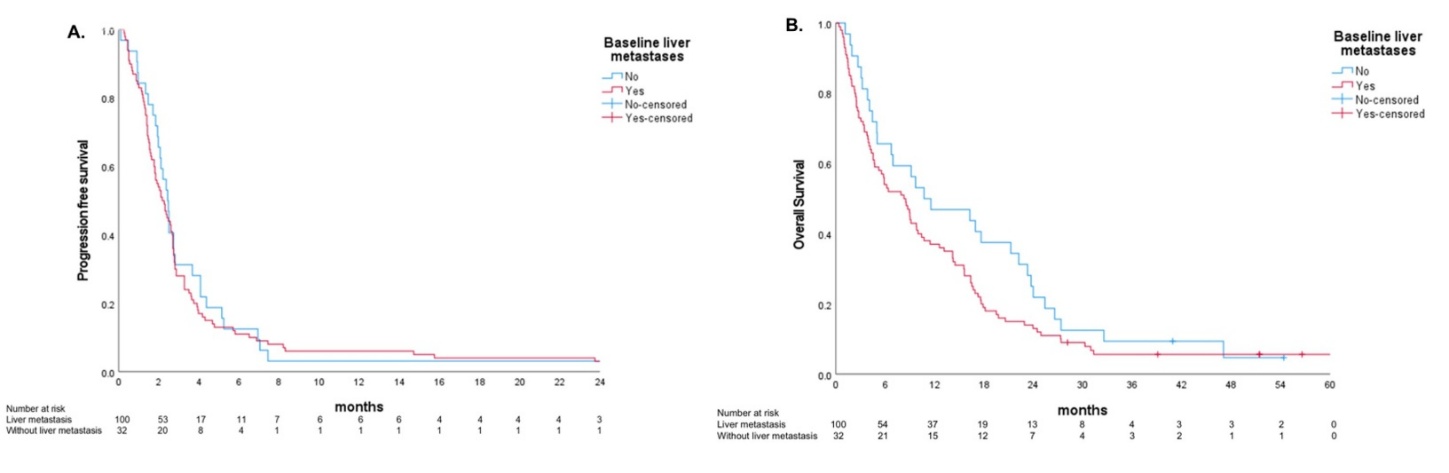
**

A. Median PFS was 2.43 months (95% CI: 2.10–2.77) in patients without liver metastases at baseline, compared to 2.17 months (95% CI: 1.68–2.66) in those with liver metastases (*P* = 0.597).
B. Median OS was 10.70 months (95% CI: 0.81–20.59) in patients without liver metastases at baseline, versus 8.27 months (95% CI: 5.45–11.09) in those with liver metastases (*P* = 0.129).
