# Supplementary material for: Effect of Liver Metastases on Survival in Microsatellite-Stable Metastatic Colorectal Cancer Treated with Immune Checkpoint Inhibitors
Source: Cancer Res Commun. 2026 Feb 18;6(2):340–9. doi: 10.1158/2767-9764.CRC-25-0690 (PMC13038315; doi:10.1158/2767-9764.CRC-25-0690)
Supplement: Supplementary Table 1 — Details of molecular characteristics and tumor mutational burden by timing of treatment [file crc-25-0690_supplementary_table_1_suppst1.docx]

**Supplementary Table 1. Details of molecular characteristics and tumor mutational burden by timing of treatment**

|  | **Baseline**  **(Treatment naïve)**  **N = 63** | **Before**  **ICI-based therapy**  **N = 86** | **After**  **ICI-based therapy**  **N = 20** |
| --- | --- | --- | --- |
| **Type of testing, n (%)**  Oncopanel  Snapshot  Guardant360  Foundation one  Multigene PCR  Others  Unknown | 33(52.4)  5(8)  2(3.2)  4(6.3)  12(19)  2(3.2)  5(7.9) | 44(51.2)  15(17.5)  16(18.6)  2(2.3)  4(4.6)  2(2.3)  3(3.5) | 3(15)  1(5)  15(75)  1(5)  0  0  0 |
| **Specimen for testing, n (%)**  Tissue  Primary tumor  Metastatic site  Plasma  Unknown | 54(85.7)  43(68.2)  11(17.5)  3(4.8)  6(9.5) | 66(76.7)  43 (50)  23(26.7)  16(18.6)  4(4.7) | 3(15)  1(5)  2(10)  17(85)  0 |
| **Molecular characteristics, n (%)**  KRAS mutation  BRAF mutation  NRAS mutation  APC mutation  TP53 mutation  PIK3CA mutation  None of these above | 37(58.7)  5(7.9)  6(9.5)  37(58.7)  41(65.1)  19(30.2)  0 | 48(55.8)  7(8.1)  6(7)  58(67.4)  68(79.1)  15(17.4)  1(1.2) | 11(55)  2(10)  5(25)  15(75)  19(95)  7(35)  1(5) |
| **TMB (mut/mb), n (%)**  < 10  10-20  >20  unknown | 18(28.6)  12(19)  0  33(52.4) | 22(25.6)  22(25.6)  5(5.8)  37(43) | 3(15)  5(25)  1(5)  11(55) |

Abbreviations: ICI, Immune checkpoint inhibitor;TMB, tumor mutational burden; mut/mb=mutations per mega base
