# Supplementary material for: Effect of Liver Metastases on Survival in Microsatellite-Stable Metastatic Colorectal Cancer Treated with Immune Checkpoint Inhibitors
Source: Cancer Res Commun. 2026 Feb 18;6(2):340–9. doi: 10.1158/2767-9764.CRC-25-0690 (PMC13038315; doi:10.1158/2767-9764.CRC-25-0690)
Supplement: Supplementary Table 2 — Regimens of immune checkpoint inhibitor-based therapies [file crc-25-0690_supplementary_table_2_suppst2.docx]

**Supplementary Table 2. Regimens of immune checkpoint inhibitor-based therapies**

| **Regimens** | **ALL**  **N = 132** | **Liver**  **metastases**  **N = 93** | **Without liver**  **metastases**  **N = 39** |
| --- | --- | --- | --- |
| **Investigational regimens, n (%)** | **101(76.5)** | **73(78.5)** | **28(71.8)** |
| - Atezolizumab and Bevacizumab and Imprime-PGG^[1]^ | 1(0.8) | 1(1.1) | 0 |
| - Atezolizumab and Cabozantinib^[2]^ | 9(6.8) | 7(7.5) | 2(5.1) |
| - Atezolizumab and Cobimetinib^[3]^ | 8(6.1) | 6(6.5) | 2(5.1) |
| - Atezolizumab and Emactuzumab^[4]^ | 2(1.5) | 2(2.2) | 0 |
| - Atezolizumab and GDC0919^[5]^ | 1(0.8) | 1(1.1) | 0 |
| - Atezolizumab and Regorafenib^[1]^ | 1(0.8) | 1(1.1) | 0 |
| - Atezolizumab and Tiragolumab^[6]^ | 4(3.0) | 4(4.3) | 0 |
| - Avelumab and Talazoparib^[7]^ | 1(0.8) | 1(1.1) | 0 |
| - Durvalumab and Cabozantinib^[8]^ | 1(0.8) | 1(1.1) | 0 |
| - Durvalumab and Monalizumab^[9]^ | 2(1.5) | 2(2.2) | 0 |
| - Durvalumab and Tremelimumab^[10]^ | 2(1.5) | 2(2.2) | 0 |
| - Nivolumab and Bevacizumab and FOLFOX^[11]^ | 2(1.5) | 2(2.2) | 0 |
| - Nivolumab and Copanlisib^[12]^ | 12(9.1) | 4(4.3) | 8(20.5) |
| - Nivolumab and Relatlimab^[13]^ | 2(1.5) | 2(2.2) | 0 |
| - Pembrolizumab and Aflibercept^[14]^ | 1(0.8) | 1(1.1) | 0 |
| - Pembrolizumab and AMG820^[15]^ | 7(5.3) | 3(3.2) | 4(10.3) |
| - Pembrolizumab and CGX1321^[16]^ | 7(5.3) | 5(5.4) | 2(5.1) |
| - Pembrolizumab and INCB001158^[17]^ | 1(0.8) | 1(1.1) | 0 |
| - Pembrolizumab and mRNA 4157^[18]^ | 4(3.0) | 4(4.3) | 0 |
| - Pembrolizumab and Trebananib^[19]^ | 33(25) | 23(24.7) | 10(25.6) |
| **Physician-selected regimens, n (%)** | **31(23.5)** | **20(21.5)** | **11(28.2)** |
| - Nivolumab | 2(1.5) | 2(2.2) | 0 |
| - Nivolumab and Cetuximab and Encorafenib | 1(0.8) | 1(1.1) | 0 |
| - Nivolumab and FOLFIRI | 1(0.8) | 1(1.1) | 0 |
| - Nivolumab and Ipilimumab | 2(1.5) | 1(1.1) | 1(2.6) |
| - Nivolumab and Regorafenib | 3(2.3) | 2(2.2) | 1(2.6) |
| - Pembrolizumab | 20(15.2) | 13(14.0) | 7(17.9) |
| - Pembrolizumab and Bevacizumab | 1(0.8) | 0 | 1(2.6) |
| - Pembrolizumab and Carboplatin and Pemetrexed | 1(0.8) | 0 | 1(2.6) |

**References**

1. Chau I, Haag GM, Rahma OE, Macarulla TM, McCune SL, Yardley DA, et al. MORPHEUS: A phase Ib/II umbrella study platform evaluating the safety and efficacy of multiple cancer immunotherapy (CIT)-based combinations in different tumour types. Annals of Oncology. 2018;29:viii439-viii40.

2. Exelixis. Study of Cabozantinib in Combination With Atezolizumab to Subjects With Locally Advanced or Metastatic Solid Tumors 2023 [updated July 27, 2023. Available from: https://clinicaltrials.gov/study/NCT03170960.

3. Hellmann MD, Kim TW, Lee CB, Goh BC, Miller WH, Jr., Oh DY, et al. Phase Ib study of atezolizumab combined with cobimetinib in patients with solid tumors. Ann Oncol. 2019;30(7):1134-42.

4. Gomez-Roca C, Cassier P, Zamarin D, Machiels JP, Perez Gracia JL, Stephen Hodi F, et al. Anti-CSF-1R emactuzumab in combination with anti-PD-L1 atezolizumab in advanced solid tumor patients naïve or experienced for immune checkpoint blockade. J Immunother Cancer. 2022;10(5).

5. Jung KH, LoRusso P, Burris H, Gordon M, Bang YJ, Hellmann MD, et al. Phase I Study of the Indoleamine 2,3-Dioxygenase 1 (IDO1) Inhibitor Navoximod (GDC-0919) Administered with PD-L1 Inhibitor (Atezolizumab) in Advanced Solid Tumors. Clin Cancer Res. 2019;25(11):3220-8.

6. Kim TW, Bedard PL, LoRusso P, Gordon MS, Bendell J, Oh DY, et al. Anti-TIGIT Antibody Tiragolumab Alone or With Atezolizumab in Patients With Advanced Solid Tumors: A Phase 1a/1b Nonrandomized Controlled Trial. JAMA Oncol. 2023;9(11):1574-82.

7. Yap TA, Bardia A, Dvorkin M, Galsky MD, Beck JT, Wise DR, et al. Avelumab Plus Talazoparib in Patients With Advanced Solid Tumors: The JAVELIN PARP Medley Nonrandomized Controlled Trial. JAMA Oncol. 2023;9(1):40-50.

8. Saeed A, Park R, Pathak H, Al-Bzour AN, Dai J, Phadnis M, et al. Clinical and biomarker results from a phase II trial of combined cabozantinib and durvalumab in patients with chemotherapy-refractory colorectal cancer (CRC): CAMILLA CRC cohort. Nat Commun. 2024;15(1):1533.

9. Patel SP, Alonso-Gordoa T, Banerjee S, Wang D, Naidoo J, Standifer NE, et al. Phase 1/2 study of monalizumab plus durvalumab in patients with advanced solid tumors. J Immunother Cancer. 2024;12(2).

10. Institute NC. Durvalumab and Tremelimumab With or Without High or Low-Dose Radiation Therapy in Treating Patients With Metastatic Colorectal or Non-small Cell Lung Cancer 2025 [updated July 4, 2025. Available from: https://clinicaltrials.gov/study/NCT02888743.

11. Lenz HJ, Parikh A, Spigel DR, Cohn AL, Yoshino T, Kochenderfer M, et al. Modified FOLFOX6 plus bevacizumab with and without nivolumab for first-line treatment of metastatic colorectal cancer: phase 2 results from the CheckMate 9X8 randomized clinical trial. J Immunother Cancer. 2024;12(3).

12. Jakubowski C, Collins NB, Sugar EA, Berg M, Cao H, Giannakis M, et al. A phase I/II study of PI3Kinase inhibition with copanlisib combined with the anti-PD-1 antibody nivolumab in relapsed/refractory solid tumors with expansions in MSS colorectal cancer. Journal of Clinical Oncology. 2020;38(15_suppl):TPS4114-TPS.

13. Christenson E, Durham JN, Brancati M, Bruning HD, Petrie S, Wang H, et al. A phase 2 study evaluating response and biomarkers in patients with microsatellite stable (MSS) advanced colorectal cancer (CRC) treated with nivolumab/relatlimab. Journal of Clinical Oncology. 2024;42(16_suppl):3554-.

14. Rahma OE, Tyan K, Giobbie-Hurder A, Brohl AS, Bedard PL, Renouf DJ, et al. Phase IB study of ziv-aflibercept plus pembrolizumab in patients with advanced solid tumors. J Immunother Cancer. 2022;10(3).

15. Razak AR, Cleary JM, Moreno V, Boyer M, Calvo Aller E, Edenfield W, et al. Safety and efficacy of AMG 820, an anti-colony-stimulating factor 1 receptor antibody, in combination with pembrolizumab in adults with advanced solid tumors. J Immunother Cancer. 2020;8(2).

16. Giannakis M, Le DT, Pishvaian MJ, Weinberg BA, Papadopoulos KP, Shen L, et al. Phase 1 study of WNT pathway Porcupine inhibitor CGX1321 and phase 1b study of CGX1321 + pembrolizumab (pembro) in patients (pts) with advanced gastrointestinal (GI) tumors. Journal of Clinical Oncology. 2023;41(16_suppl):3514-.

17. Naing A, Papadopoulos KP, Pishvaian MJ, Rahma O, Hanna GJ, Garralda E, et al. First-in-human phase 1 study of the arginase inhibitor INCB001158 alone or combined with pembrolizumab in patients with advanced or metastatic solid tumours. BMJ Oncol. 2024;3(1):e000249.

18. Julie B, Howard B, Jeffrey C, Manish P, Daniel C, Martin G, et al. 798 Safety, tolerability, and immunogenicity of mRNA-4157 in combination with pembrolizumab in subjects with unresectable solid tumors (KEYNOTE-603): an update. Journal for ImmunoTherapy of Cancer. 2020;8(Suppl 3).

19. Huffman BM, Rahma OE, Tyan K, Li YY, Giobbie-Hurder A, Schlechter BL, et al. A Phase I Trial of Trebananib, an Angiopoietin 1 and 2 Neutralizing Peptibody, Combined with Pembrolizumab in Patients with Advanced Ovarian and Colorectal Cancer. Cancer Immunol Res. 2025;13(1):9-22.
