# Supplementary material for: Effect of Liver Metastases on Survival in Microsatellite-Stable Metastatic Colorectal Cancer Treated with Immune Checkpoint Inhibitors
Source: Cancer Res Commun. 2026 Feb 18;6(2):340–9. doi: 10.1158/2767-9764.CRC-25-0690 (PMC13038315; doi:10.1158/2767-9764.CRC-25-0690)
Supplement: Supplementary Table 3 — Subsequent treatments after immune checkpoint inhibitor-based therapy [file crc-25-0690_supplementary_table_3_suppst3.docx]

**Supplementary Table 3. Subsequent treatments after immune checkpoint inhibitor-based**

| **Regimens** | **ALL**  **N = 132** | **Liver metastases**  **N = 93** | **Without liver**  **metastases**  **N = 39** |
| --- | --- | --- | --- |
| Patients who received subsequent therapy, n (%) | 63 (47.7) | 42 (45.2) | 21 (53.8) |
| FOLFOX/FOLFIRI | 40 (30.3) | 29 (31.2) | 11 (28.2) |
| Anti-VEGF | 32 (24.2) | 21 (22.6) | 11 (28.2) |
| Anti-EGFR | 11 (8.3) | 8 (8.6) | 3 (7.7) |
| BRAF/MEK inhibitor | 3 (2.3) | 2 (2.2) | 1 (2.6) |
| Trifluridine/Tipiracil | 28 (21.2) | 16 (17.2) | 12 (30.8) |
| Regorafenib | 12 (9.1) | 9 (9.7) | 3 (7.7) |
| Fruquintinib | 2 (1.5) | 1 (1.1) | 1 (2.6) |
| HER2 targeted therapies | 3 (2.3) | 1 (1.1) | 2 (5.1) |
| Other targeted therapies | 2 (1.5) | 2 (2.2) | 0 |
| Clinical trials | 6 (4.5) | 4 (4.3) | 2 (5.1) |
