# Supplementary material for: Effect of Liver Metastases on Survival in Microsatellite-Stable Metastatic Colorectal Cancer Treated with Immune Checkpoint Inhibitors
Source: Cancer Res Commun. 2026 Feb 18;6(2):340–9. doi: 10.1158/2767-9764.CRC-25-0690 (PMC13038315; doi:10.1158/2767-9764.CRC-25-0690)
Supplement: Supplementary Table 4 — Sites of progressive disease during immune checkpoint inhibitor-based therapy [file crc-25-0690_supplementary_table_4_suppst4.docx]

**Supplementary Table 4. Sites of progressive disease during immune checkpoint inhibitor-based therapy**

| **Sites, n (%)** | **ALL**  **N = 132** | **Liver**  **metastases**  **N = 93** | **Without liver**  **metastases**  **N = 39** | **P-value** |
| --- | --- | --- | --- | --- |
| **Liver** | **83 (62.9)** | **80 (86)** | **3 (7.7)** | **<0.001** |
| Lymph nodes | 70 (53) | 52 (55.9) | 18 (46.2) | 0.305 |
| Lung | 75 (56.8) | 55 (59.1) | **20 (51.3)** | 0.406 |
| Peritoneum | 35 (26.5) | 21 (22.6) | 14 (35.9) | 0.114 |
| Bone | 19 (14.4) | 11 (11.8) | 8 (20.5) | 0.195 |
| Others | 27 (20.5) | 15 (16.1) | 12 (30.8) | 0.057 |
